# 2-(1,3-Dioxoisoindolin-2-yl)propanoic acid

**DOI:** 10.1107/S1600536809040434

**Published:** 2009-10-10

**Authors:** Abdul Rauf Raza, Aisha Saddiqa, M. Nawaz Tahir, Muhammad Danish, Talat Majeed

**Affiliations:** a Department of Chemistry, University of Sargodha, Sargodha, Pakistan; b Department of Physics, University of Sargodha, Sargodha, Pakistan

## Abstract

The crystal structure of the title compound, C_11_H_9_NO_4_, consists of infinite one-dimensional polymeric chains due to inter­molecular O—H⋯O hydrogen bonds between the carboxyl­ate and carbonyl groups. The phthalimide ring system and the C—COO group are planar, with r.m.s. deviations of 0.0253 and 0.0067 Å, respectively, from their mean square planes and the dihedral angle between them is 66.41 (7)°. The mol­ecules are stabilized by C=O⋯π inter­actions and weak intra­molecular C—H⋯O hydrogen bonds.

## Related literature

For the medicinal properties of isocoumarin, see: Matsuda *et al.* (1999 ▶). For related crystal structures, see: Li & Liang (2006 ▶); Raza *et al.* (2009 ▶); Wheeler *et al.* (2004 ▶). For the graph-set analysis of hydrogen-bond patterns in crystal structures, see: Bernstein *et al.* (1995 ▶).
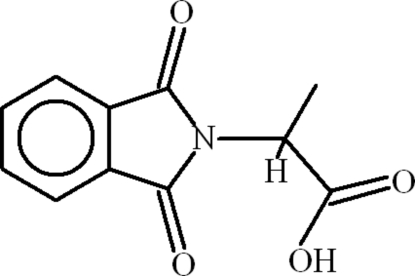


## Experimental

### 

#### Crystal data

C_11_H_9_NO_4_
*M* *_r_* = 219.19
Monoclinic, 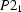

*a* = 9.3056 (8) Å
*b* = 5.9768 (4) Å
*c* = 9.7583 (8) Å
β = 110.988 (3)°
*V* = 506.73 (7) Å^3^
*Z* = 2
Mo *K*α radiation
μ = 0.11 mm^−1^
*T* = 296 K
0.30 × 0.25 × 0.23 mm

#### Data collection

Bruker Kappa APEXII CCD diffractometer
Absorption correction: multi-scan (*SADABS*; Bruker, 2005 ▶) *T*_min_ = 0.968, *T*_max_ = 0.974
5705 measured reflections
1381 independent reflections
1302 reflections with *I* > 2σ(*I*)
*R*_int_ = 0.023

#### Refinement

*R*[*F* ^2^ > 2σ(*F* ^2^)] = 0.030
*wR*(*F* ^2^) = 0.082
*S* = 1.06
1381 reflections
153 parameters
1 restraint
H atoms treated by a mixture of independent and constrained refinement
Δρ_max_ = 0.16 e Å^−3^
Δρ_min_ = −0.13 e Å^−3^

### 

Data collection: *APEX2* (Bruker, 2007 ▶); cell refinement: *SAINT* (Bruker, 2007 ▶); data reduction: *SAINT*; program(s) used to solve structure: *SHELXS97* (Sheldrick, 2008 ▶); program(s) used to refine structure: *SHELXL97* (Sheldrick, 2008 ▶); molecular graphics: *ORTEP-3 for Windows* (Farrugia, 1997 ▶) and *PLATON* (Spek, 2009 ▶); software used to prepare material for publication: *WinGX* (Farrugia, 1999 ▶) and *PLATON*.

## Supplementary Material

Crystal structure: contains datablocks global, I. DOI: 10.1107/S1600536809040434/si2209sup1.cif

Structure factors: contains datablocks I. DOI: 10.1107/S1600536809040434/si2209Isup2.hkl

Additional supplementary materials:  crystallographic information; 3D view; checkCIF report

## Figures and Tables

**Table 1:** Hydrogen-bond geometry (Å, °)

*D*—H⋯*A*	*D*—H	H⋯*A*	*D*⋯*A*	*D*—H⋯*A*
O3—H3*A*⋯O1^i^	0.80 (4)	1.96 (4)	2.750 (2)	172 (4)
C9—H9⋯O2	0.96 (3)	2.48 (2)	2.899 (2)	106.6 (17)
C8—O2⋯*Cg*1^ii^	1.20 (1)	3.09 (1)	4.0543 (17)	138 (1)

Symmetry codes: (i) 
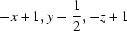
; (ii) 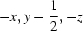. *Cg*1 is the centroid of 5-membered heterocyclic ring.

**Fig. 1.**
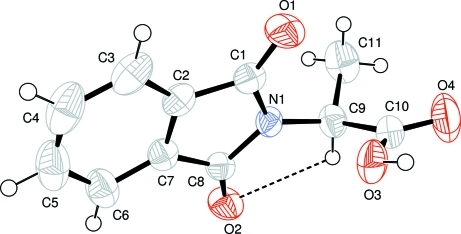
View of (I) with the atom numbering scheme. The thermal ellipsoids are drawn at the 50% probability level. H-atoms are shown by small circles of arbitrary radii. The dotted line represent the intramolecular H-bonding.

**Fig. 2.**
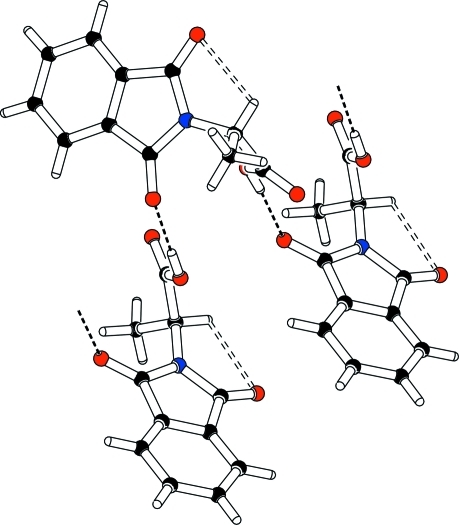
The partial packing (PLATON; Spek, 2009) which shows that molecules form infinite one dimensional polymeric chains.

## supplementary crystallographic information

### Comment

Isocoumarin are important components among natural products that exhibit a broad range of biological activities including anti-microbial, anti-allergic and immunomodulatory (Matsuda *et al.*, 1999). Isocoumarins are also useful intermediates in synthesis of many important compounds *e.g.*, isoquinoline alkaloids. The titled compound (I, Fig. 1), is an intermediate towards the synthesis of isocoumarins. The title compound has also been prepared for complexation with various metals.

We have recently reported the crystal structure of (II) (2*R*)-2-(1,3-Dioxoisoindolin-2-yl)-4-(methylsulfanyl)butanoic acid (Raza *et al.*, 2009) which contain the same isoindoline. The crystal structures of (III) DL-2-(1,3-Dioxoisoindolin-2-yl)propanoic acid (Wheeler *et al.*, 2004), (IV) (*S*)-2-(1,3-Dioxoisoindolin-2-yl)propanoic acid (Li & Liang, 2006) have also been reported which are the racemate of (I).

In (I) the phthalimide ring system A (C1—C8/N1/O1/O2) and the group B (C9/C10/O3/O4) are planar with r.m.s. deviations of 0.0253 and 0.0067 Å respectively, from their mean square planes. The dihedral angle between A/B is 66.41 (7)°, whereas it is 86.7 (3)° as observed in (IV). The title compound is stabilized in the form of infinite one dimensional polymeric chains due to intermolecular H-bondings (Table 1, Fig. 2). There exist a weak intramolecular H-bondings forming S(5) ring motifs (Fig. 1) (Bernstein *et al.*, 1995). The C==O···*Cg*1 [*Cg*1 is the centroid of five membered ring (C1/C2/C7/C8/N1)] interaction (Table 1), may also be responsible for stabilizing of the molecules.

### Experimental

The (*S*)-alanine (1.96 g, 22 mmol) and phthallic anhydride (3.6 g, 24.3 mmol) were added to a flask with constant stirring. The temperature of oil bath was kept at 433 K. Three hours later the flask was removed from oil bath, brought to room temperature and the crystals of phthallic anhydride on the walls of the flask were removed manually. The solid crude product was purified by crystallization from ethanol:water (8:2) that yielded (70%) colorless prisms of the title compound (I).

### Refinement

In the absence of significant anomalous dispersion effects, Friedel pairs were averaged using MERG 3.

The coordinates of H3A and H9 were refined. The H-atoms were positioned geometrically with C—H = 0.93 and 0.96 Å for aromatic and methyl H atoms respectively and constrained to ride on their parent atoms, with *U*_iso_(H) = *xU*_eq_(C, O), where *x* = 1.5 for methyl and 1.2 for all other H atoms.

### Crystal data

**Table:**

C_11_H_9_NO_4_	*F*(000) = 228
*M**_r_* = 219.19	*D*_x_ = 1.437 Mg m^−^^3^
Monoclinic, *P*2_1_	Mo *K*α radiation, λ = 0.71073 Å
Hall symbol: P 2yb	Cell parameters from 2319 reflections
*a* = 9.3056 (8) Å	θ = 2.2–28.3°
*b* = 5.9768 (4) Å	µ = 0.11 mm^−^^1^
*c* = 9.7583 (8) Å	*T* = 296 K
β = 110.988 (3)°	Prism, colorless
*V* = 506.73 (7) Å^3^	0.30 × 0.25 × 0.23 mm
*Z* = 2	

### Data collection

**Table:**

Bruker Kappa APEXII CCD diffractometer	1381 independent reflections
Radiation source: fine-focus sealed tube	1302 reflections with *I* > 2σ(*I*)
graphite	*R*_int_ = 0.023
Detector resolution: 7.40 pixels mm^-1^	θ_max_ = 28.3°, θ_min_ = 2.2°
ω scans	*h* = −12→12
Absorption correction: multi-scan (*SADABS*; Bruker, 2005)	*k* = −7→7
*T*_min_ = 0.968, *T*_max_ = 0.974	*l* = −12→13
5705 measured reflections	

### Refinement

**Table:**

Refinement on *F*^2^	Secondary atom site location: difference Fourier map
Least-squares matrix: full	Hydrogen site location: inferred from neighbouring sites
*R*[*F*^2^ > 2σ(*F*^2^)] = 0.030	H atoms treated by a mixture of independent and constrained refinement
*wR*(*F*^2^) = 0.082	*w* = 1/[σ^2^(*F*_o_^2^) + (0.0443*P*)^2^ + 0.0584*P*] where *P* = (*F*_o_^2^ + 2*F*_c_^2^)/3
*S* = 1.06	(Δ/σ)_max_ < 0.001
1381 reflections	Δρ_max_ = 0.16 e Å^−^^3^
153 parameters	Δρ_min_ = −0.13 e Å^−^^3^
1 restraint	Extinction correction: *SHELXL97* (Sheldrick, 2008)
Primary atom site location: structure-invariant direct methods	Extinction coefficient: 0.132 (13)

### Special details

**Table:**

Geometry. Bond distances, angles *etc*. have been calculated using the rounded fractional coordinates. All su's are estimated from the variances of the (full) variance-covariance matrix. The cell e.s.d.'s are taken into account in the estimation of distances, angles and torsion angles
Refinement. Refinement of *F*^2^ against ALL reflections. The weighted *R*-factor *wR* and goodness of fit *S* are based on *F*^2^, conventional *R*-factors *R* are based on *F*, with *F* set to zero for negative *F*^2^. The threshold expression of *F*^2^ > σ(*F*^2^) is used only for calculating *R*-factors(gt) *etc*. and is not relevant to the choice of reflections for refinement. *R*-factors based on *F*^2^ are statistically about twice as large as those based on *F*, and *R*- factors based on ALL data will be even larger.

### Fractional atomic coordinates and isotropic or equivalent isotropic displacement parameters (Å^2^)

**Table:**

	*x*	*y*	*z*	*U*_iso_*/*U*_eq_	
O1	0.34281 (15)	1.0490 (3)	0.31137 (15)	0.0551 (4)	
O2	−0.00322 (14)	0.5031 (3)	0.09136 (15)	0.0520 (4)	
O3	0.41497 (15)	0.5761 (3)	0.42520 (15)	0.0550 (5)	
O4	0.57532 (16)	0.5164 (5)	0.30695 (19)	0.0817 (7)	
N1	0.19830 (14)	0.7462 (3)	0.19714 (14)	0.0358 (4)	
C1	0.22202 (19)	0.9473 (3)	0.27082 (17)	0.0382 (4)	
C2	0.07493 (19)	1.0033 (3)	0.28948 (17)	0.0402 (5)	
C3	0.0385 (3)	1.1806 (4)	0.3606 (2)	0.0562 (6)	
C4	−0.1092 (3)	1.1832 (5)	0.3656 (2)	0.0690 (8)	
C5	−0.2125 (3)	1.0146 (6)	0.3031 (2)	0.0684 (9)	
C6	−0.1755 (2)	0.8353 (5)	0.2313 (2)	0.0539 (6)	
C7	−0.02955 (19)	0.8354 (3)	0.22485 (16)	0.0392 (4)	
C8	0.04667 (17)	0.6700 (3)	0.16030 (16)	0.0361 (4)	
C9	0.31769 (19)	0.6226 (4)	0.1658 (2)	0.0432 (5)	
C10	0.45173 (19)	0.5679 (4)	0.3068 (2)	0.0470 (5)	
C11	0.3710 (3)	0.7438 (6)	0.0556 (2)	0.0642 (8)	
H3	0.10903	1.29359	0.40332	0.0674*	
H3A	0.490 (4)	0.564 (6)	0.497 (4)	0.0660*	
H4	−0.13859	1.30102	0.41199	0.0828*	
H5	−0.30990	1.02072	0.30905	0.0820*	
H6	−0.24537	0.72111	0.18964	0.0647*	
H9	0.273 (2)	0.482 (5)	0.125 (2)	0.0518*	
H11A	0.28411	0.77266	−0.03225	0.0962*	
H11B	0.44407	0.65239	0.03244	0.0962*	
H11C	0.41850	0.88289	0.09692	0.0962*	

### Atomic displacement parameters (Å^2^)

**Table:**

	*U*^11^	*U*^22^	*U*^33^	*U*^12^	*U*^13^	*U*^23^
O1	0.0464 (7)	0.0507 (8)	0.0545 (7)	−0.0167 (7)	0.0015 (5)	−0.0070 (7)
O2	0.0474 (7)	0.0484 (8)	0.0563 (7)	−0.0153 (6)	0.0139 (6)	−0.0153 (7)
O3	0.0409 (6)	0.0693 (10)	0.0459 (7)	0.0152 (7)	0.0046 (5)	0.0073 (7)
O4	0.0412 (7)	0.1193 (17)	0.0869 (11)	0.0252 (10)	0.0257 (8)	0.0306 (13)
N1	0.0288 (6)	0.0363 (7)	0.0363 (6)	0.0000 (5)	0.0042 (5)	−0.0024 (6)
C1	0.0385 (8)	0.0368 (8)	0.0310 (7)	−0.0025 (7)	0.0024 (6)	−0.0004 (6)
C2	0.0470 (9)	0.0382 (9)	0.0311 (7)	0.0044 (7)	0.0088 (6)	0.0021 (7)
C3	0.0773 (13)	0.0452 (11)	0.0407 (8)	0.0125 (10)	0.0147 (9)	−0.0034 (8)
C4	0.0893 (16)	0.0758 (17)	0.0461 (10)	0.0356 (15)	0.0293 (10)	0.0019 (11)
C5	0.0612 (12)	0.098 (2)	0.0532 (11)	0.0276 (14)	0.0292 (10)	0.0102 (13)
C6	0.0444 (9)	0.0727 (14)	0.0478 (9)	0.0048 (10)	0.0203 (8)	0.0056 (10)
C7	0.0382 (7)	0.0451 (9)	0.0314 (7)	0.0023 (7)	0.0089 (6)	0.0036 (7)
C8	0.0327 (7)	0.0384 (8)	0.0325 (7)	−0.0032 (7)	0.0060 (5)	0.0005 (6)
C9	0.0346 (8)	0.0473 (10)	0.0444 (8)	0.0009 (8)	0.0102 (6)	−0.0067 (8)
C10	0.0338 (7)	0.0475 (10)	0.0566 (10)	0.0031 (8)	0.0126 (7)	0.0069 (9)
C11	0.0556 (11)	0.0928 (18)	0.0468 (10)	0.0056 (13)	0.0217 (8)	0.0027 (12)

### Geometric parameters (Å, °)

**Table:**

O1—C1	1.213 (2)	C5—C6	1.390 (4)
O2—C8	1.200 (2)	C6—C7	1.382 (3)
O3—C10	1.318 (2)	C7—C8	1.482 (2)
O4—C10	1.190 (3)	C9—C10	1.525 (3)
O3—H3A	0.80 (4)	C9—C11	1.520 (3)
N1—C8	1.402 (2)	C3—H3	0.9300
N1—C9	1.455 (3)	C4—H4	0.9300
N1—C1	1.377 (2)	C5—H5	0.9300
C1—C2	1.482 (3)	C6—H6	0.9300
C2—C3	1.374 (3)	C9—H9	0.96 (3)
C2—C7	1.382 (2)	C11—H11A	0.9600
C3—C4	1.393 (4)	C11—H11B	0.9600
C4—C5	1.375 (4)	C11—H11C	0.9600
			
O1···O3	3.023 (2)	C5···C11^viii^	3.551 (3)
O1···C10	3.055 (3)	C6···O4^ix^	3.283 (3)
O1···C10^i^	3.268 (3)	C7···O2^viii^	3.361 (2)
O1···C11	3.177 (3)	C8···O2^viii^	3.074 (2)
O1···O3^ii^	2.750 (2)	C8···C3^iv^	3.534 (3)
O2···C7^iii^	3.361 (2)	C10···O1	3.055 (3)
O2···N1^iii^	3.149 (2)	C10···O1^iv^	3.268 (3)
O2···C3^iv^	3.168 (3)	C11···C5^iii^	3.551 (3)
O2···C8^iii^	3.074 (2)	C11···O1	3.177 (3)
O2···C1^iii^	3.405 (2)	C1···H3A^ii^	2.91 (4)
O3···N1	2.615 (2)	C1···H11C	2.9300
O3···O1	3.023 (2)	C2···H4^x^	3.0100
O3···C1	2.912 (2)	C3···H4^x^	3.0800
O3···O1^v^	2.750 (2)	C5···H3^x^	2.9800
O4···C6^vi^	3.283 (3)	C5···H11A^viii^	2.9200
O1···H3A^ii^	1.96 (4)	H3···C5^xi^	2.9800
O1···H11C	2.6300	H3A···O1^v^	1.96 (4)
O2···H11A^iii^	2.8300	H3A···C1^v^	2.91 (4)
O2···H9	2.48 (2)	H4···O4^xii^	2.8000
O4···H4^vii^	2.8000	H4···C2^xi^	3.0100
O4···H6^vi^	2.6400	H4···C3^xi^	3.0800
O4···H11B	2.6400	H6···O4^ix^	2.6400
N1···O3	2.615 (2)	H9···O2	2.48 (2)
N1···O2^viii^	3.149 (2)	H11A···O2^viii^	2.8300
C1···O3	2.912 (2)	H11A···C5^iii^	2.9200
C1···O2^viii^	3.405 (2)	H11B···O4	2.6400
C3···C8^i^	3.534 (3)	H11C···O1	2.6300
C3···O2^i^	3.168 (3)	H11C···C1	2.9300
			
C10—O3—H3A	110 (3)	N1—C9—C10	111.01 (15)
C1—N1—C9	124.14 (15)	O3—C10—C9	113.43 (16)
C8—N1—C9	123.75 (16)	O4—C10—C9	122.29 (18)
C1—N1—C8	112.08 (15)	O3—C10—O4	124.24 (19)
O1—C1—C2	129.45 (17)	C2—C3—H3	122.00
N1—C1—C2	106.19 (15)	C4—C3—H3	122.00
O1—C1—N1	124.35 (17)	C3—C4—H4	119.00
C1—C2—C3	129.93 (19)	C5—C4—H4	119.00
C3—C2—C7	122.00 (19)	C4—C5—H5	119.00
C1—C2—C7	108.03 (15)	C6—C5—H5	119.00
C2—C3—C4	116.8 (2)	C5—C6—H6	122.00
C3—C4—C5	121.2 (3)	C7—C6—H6	122.00
C4—C5—C6	121.9 (3)	N1—C9—H9	106.4 (13)
C5—C6—C7	116.6 (2)	C10—C9—H9	106.2 (13)
C2—C7—C6	121.44 (18)	C11—C9—H9	109.1 (12)
C2—C7—C8	108.22 (16)	C9—C11—H11A	109.00
C6—C7—C8	130.30 (18)	C9—C11—H11B	109.00
O2—C8—N1	124.46 (16)	C9—C11—H11C	109.00
O2—C8—C7	130.15 (16)	H11A—C11—H11B	109.00
N1—C8—C7	105.39 (14)	H11A—C11—H11C	109.00
N1—C9—C11	112.0 (2)	H11B—C11—H11C	109.00
C10—C9—C11	111.83 (17)		
			
C8—N1—C1—O1	−178.37 (16)	C1—C2—C7—C6	176.60 (16)
C8—N1—C1—C2	2.54 (18)	C1—C2—C7—C8	−1.21 (18)
C9—N1—C1—O1	3.8 (3)	C3—C2—C7—C6	−1.4 (3)
C9—N1—C1—C2	−175.31 (15)	C3—C2—C7—C8	−179.17 (16)
C1—N1—C8—O2	177.22 (16)	C2—C3—C4—C5	0.5 (3)
C1—N1—C8—C7	−3.24 (18)	C3—C4—C5—C6	−0.5 (4)
C9—N1—C8—O2	−4.9 (3)	C4—C5—C6—C7	−0.4 (3)
C9—N1—C8—C7	174.62 (15)	C5—C6—C7—C2	1.3 (3)
C1—N1—C9—C10	57.8 (2)	C5—C6—C7—C8	178.61 (18)
C1—N1—C9—C11	−67.9 (2)	C2—C7—C8—O2	−177.83 (18)
C8—N1—C9—C10	−119.78 (18)	C2—C7—C8—N1	2.66 (17)
C8—N1—C9—C11	114.5 (2)	C6—C7—C8—O2	4.6 (3)
O1—C1—C2—C3	−2.0 (3)	C6—C7—C8—N1	−174.88 (18)
O1—C1—C2—C7	−179.76 (18)	N1—C9—C10—O3	20.4 (3)
N1—C1—C2—C3	177.02 (18)	N1—C9—C10—O4	−161.9 (3)
N1—C1—C2—C7	−0.73 (18)	C11—C9—C10—O3	146.2 (2)
C1—C2—C3—C4	−177.09 (18)	C11—C9—C10—O4	−36.0 (4)
C7—C2—C3—C4	0.4 (3)		

Symmetry codes: (i) *x*, *y*+1, *z*; (ii) −*x*+1, *y*+1/2, −*z*+1; (iii) −*x*, *y*−1/2, −*z*; (iv) *x*, *y*−1, *z*; (v) −*x*+1, *y*−1/2, −*z*+1; (vi) *x*+1, *y*, *z*; (vii) *x*+1, *y*−1, *z*; (viii) −*x*, *y*+1/2, −*z*; (ix) *x*−1, *y*, *z*; (x) −*x*, *y*−1/2, −*z*+1; (xi) −*x*, *y*+1/2, −*z*+1; (xii) *x*−1, *y*+1, *z*.

### Hydrogen-bond geometry (Å, °)

**Table:**

*D*—H···*A*	*D*—H	H···*A*	*D*···*A*	*D*—H···*A*
O3—H3A···O1^v^	0.80 (4)	1.96 (4)	2.750 (2)	172 (4)
C9—H9···O2	0.96 (3)	2.48 (2)	2.899 (2)	106.6 (17)
C8—O2···Cg1^iii^	1.200 (2)	3.0874 (16)	4.0543 (17)	138.28 (12)

Symmetry codes: (v) −*x*+1, *y*−1/2, −*z*+1; (iii) −*x*, *y*−1/2, −*z*.
